# A single 24 h recall overestimates exclusive breastfeeding practices among infants aged less than six months in rural Ethiopia

**DOI:** 10.1186/s13006-017-0126-9

**Published:** 2017-08-01

**Authors:** Esete Habtemariam Fenta, Robel Yirgu, Bilal Shikur, Seifu Hagos Gebreyesus

**Affiliations:** Department of Reproductive Health and Health Service Management, School of Public Health, College of Health Sciences, Addis Ababa University, Addis Ababa, Ethiopia

**Keywords:** Exclusive breastfeeding, Accuracy, 24 h recall, Overestimation, IYCF

## Abstract

**Background:**

Exclusive breastfeeding (EBF) to six months is one of the World Health Organization’s (WHOs) infant and young child feeding (IYCF) core indicators. Single 24 h recall method is currently in use to measure exclusive breastfeeding practice among children of age less than six months. This approach overestimates the prevalence of EBF, especially among small population groups. This justifies the need to look for alternative measurement techniques to have a valid estimate regardless of population characteristics.

**Method:**

The study involved 422 infants of age less than six months, living in Gurage zone, Southern Ethiopia. The study was conducted from January to February 2016. Child feeding practices were measured for seven consecutive days using 24 h recall method. Recall since birth, was used to measure breastfeeding practices from birth to the day of data collection. Data on EBF obtained by using single 24 h recall were compared with seven days repeated 24 h recall method. McNemar’s test was done to assess if a significant difference existed in rates of EBF between measurement methods.

**Result:**

The mean age of infants in months was 3 (SD −1.43). Exclusive breastfeeding prevalence was highest (76.7%; 95% CI 72.6, 80.8) when EBF was estimated using single 24 h recall. The prevalence of EBF based on seven repeated 24 h recall was 53.2% (95% CI: 48.3, 58.0). The estimated prevalence of EBF since birth based on retrospective data (recall since birth) was 50.2% (95% CI 45.4, 55.1). Compared to the EBF estimates obtained from seven repeated 24 h recall, single 24 h recall overestimated EBF magnitude by 23 percentage points (95% CI 19.2, 27.8). As the number of days of 24 h recall increased, a significant decrease in overestimation of EBF was observed.

**Conclusion:**

A significant overestimation was observed when single 24 h recall was used to estimate prevalence of EBF compared to seven days of 24 h recall. By increasing the observation days we can significantly decrease the degree of overestimation. Recall since birth presented estimates of EBF that is close to seven repeated 24 h recall. This suggests that a week recall could be an alternative indicator to single 24 h recall.

## Background

Appropriate feeding practices during early infancy have a significant impact on physical growth, mental development and health of a child [1]. Among these practices exclusive breastfeeding (EBF) plays a crucial role in child health, growth and development [2]. As a result, the World Health Organization (WHO) recommends exclusive breastfeeding for the first six months after birth [3]. Suboptimal breastfeeding practices are associated with increased risk of child morbidity and mortality [2]. In developing countries, suboptimal breastfeeding is responsible for 45% of neonatal infectious death, 30% of diarrheal deaths and 18% of acute respiratory deaths among under-five children [4]. Furthermore, more than three quarters of the burden of suboptimal breastfeeding is associated with nonexclusive breastfeeding for the first six months of life [5].

The WHO has developed a number of indicators to measure infant and young child feeding practices [6]. These indicators are important both for programmatic and research purposes [1]. The prevalence of EBF among children of age less than six months is one of the core WHO indicators [6]. This particular indicator evaluates the feeding practice of an infant based on the information regarding the past 24 h only [6]. Measuring feeding practice based on a one day experience doesn’t capture the usual feeding pattern of an infant [7]. This indicator might lead to an overestimation and could misclassify an infant as exclusively breastfed where in fact the usual feeding history might indicate otherwise [8]. This misclassification may lead to a wrong conclusion that EBF has been achieved, minimizing the needed effort towards improving EBF practices [9, 10].

The WHO has acknowledged that the indicator might overestimate the prevalence of EBF [11]. Several studies have tried to validate the indicator but most have not showed the degree of variation in overestimation when repeated 24 h recall was used [9, 10, 12–21]. In our study, we examined the degree of over estimation of EBF prevalence when single 24 h recall is used as compared to seven days repeated 24 h recalls.

## Methods

### Study area

The study was conducted in Gurage zone, Meskan, Mareko and part of Silti districts. The zone is located 130 kms south of Addis Ababa, the capital city of Ethiopia. The study districts house the Demographic Surveillance System (DSS) of Addis Ababa University, under Butajira Rural Health Program of the School of Public Health. It is comprised of one urban and nine rural kebeles (the smallest administrative unit). The DSS undertakes routine data collection on vital demographic events.

### Study design and period

A community based cross-sectional study was conducted from January to February 2016.

#### Sample size and sampling procedure

Single population proportion formula was used with; 95% level of confidence, 5% margin of error, 1.5 design effect and with an estimated EBF prevalence of 52% [22] was employed to estimate the sample size, yielding 422 mother infant pairs. Households with children of age less than six months were filtered out from Butajira DSS data registry to form a sampling frame. From this frame we randomly selected 422 households with children less than six months of age.

#### Data collection process

Data was collected by using a standard interview questionnaire adapted from the Ethiopian Demographic and Health Survey. A range of sociodemographic and child health care related variables were included. We used household level ownership of fixed assets such as farm land and ownership of domestic animals to estimate household wealth.

Data were collected from mothers through a face-to-face interview for seven consecutive days. On the first day, we collected data on child characteristics, maternal demographic and socioeconomic characteristics, in addition to data on child feeding practices. On the subsequent days, we collected data only related to child feeding practices.

We employed the WHO’s 24 h recall standard itemized check list consisting of common foods developed to measure infant feeding practice. We used both 24 h recall and recall since birth methods to determine infant feeding practices. The 24 h recall interviews were administered to each visited household for seven consecutive days. At the seventh day of the interview, we assessed EBF using recall since birth (to current age). This is obtained by using an itemized check list consisting of common foods. EBF was measured by asking whether any of the foods or fluids, from the itemized checklist, were introduced at any point from birth to current age [21]. If the mother admitted introducing food or fluid, the child would be categorized as non-exclusively breastfed.

#### Data analysis procedure

We used EpiData version 3·1 for data entry and the statistical software package Stata version 11·0 for data cleaning and analysis. In the univariate analysis frequency and proportion of socio-demographic characteristics and breastfeeding practices were calculated. For continuous data, mean value of the observation and the respective standard deviation was calculated. The EBF prevalence was estimated taking all infants of age less than six months who received nothing but breast milk with the exception of medicine and oral rehydration salt as a numerator and all infants less than six months of age as a denominator.We estimated the prevalence of EBF using the following three recall methods: single 24 h recall, seven consecutive days of 24 h recall data and recall since birth. The last day (seventh day) interview data were used to determine EBF based on a single 24 h recall. We have taken the last day interview as a single 24 h recall data to help us simulate whether one day could represent an infant’s entire feeding history. These data tell us if the last day of the recall was representative of the week’s feeding history of the infant.

The multiple 24 h recall is a composite measure of EBF practices derived from more than one day interview and includes the following measures. Two consecutive 24 h recall was estimated from EBF practices on the seventh and the sixth day of interview. Three consecutive 24 h recall was estimated from EBF practices on the seventh, the sixth and the fifth day of interview. These analyses were further disaggregated by age to 2, 4 and 6 month old children.

McNemar’s test was used in order to evaluate the differences in the prevalence of EBF practices obtained from the different recall methods. Analyses were deemed statistically significant if the *p* - value is less than 0.05.

### Household wealth

Household wealth was assessed by constructing an index using principal component analysis (PCA). The domains to construct the model include characteristics of the house including floor, wall, roof, type of toilet facility, source of water, ownership of fixed assets like television, mobile, phone, refrigerator, clock, bed, main source of fuel for cooking, ownership of agricultural land, ownership of an animal including goat, chicken, sheep, donkey, ox, and cow. Cut off points were used to divide the data in to five equal groups resulting in quintiles representing the poorest to the richest households.

## Results

A total of 422 infant mother pairs were included in the study. Complete data were obtained from 410 infant mother pairs, yielding a response rate of 97.6%. Nearly equal numbers of male (213) and female (199) children participated in the study. The mean age of infants in months was three (SD = 1.43) and ranged from 0 to <6 months. Most of the respondents (99.5%) were married. The mean age of mothers in year was 28.4 (± 6 SD). The majority (76.9%) were Muslim. Occupationally, 82.5% were housewives and 9.5% were merchants.

Table 1 shows the differences in EBF prevalence estimates derived from the single, multiple 24 h recall and recall since birth and using the seven days repeated 24 h recall as reference. EBF prevalence was the highest 76.7% (95% CI 72.6, 80.8), when it was estimated using the single 24 h recall followed by EBF based on two consecutive 24 h recall 66.5% (95% CI 61.9, 71.1). The prevalence estimates sequentially decreased as the number of repeated 24 h recalls increased. The EBF prevalence based on seven repeated 24 h recall was 53.2% (95% CI 48.3, 58.0). The estimated prevalence of EBF since birth based on retrospective data (recall since birth) was 50.2%, (95% CI: 45.4, 55.1) (Table 1).

**Table 1:** Patterns of changes in EBF prevalence among infants aged 0 - < 6 months using single, multiple 24 h recall and recall since birth by using 7 repeated 24 h recall as reference, Butajira, Ethiopia (*n* = 412)

Number of 24 h recall	EBF percent (95% CI)	7 repeated 24 h recall (95% CI)	% of overestimation/under estimation (95% CI)	McNemar’s *p* - value
Single 24 h recall	76.7% (72.6, 80.8)	53.2% (48.3, 58)	23.6% (19.2, 27.8)	< 0.001*
Two consecutive 24 h recalls	66.5% (61.9, 71.1)	53.2% (48.3, 58)	13.3% (9.8, 16.9)	< 0.001*
Three consecutive 24 h recalls	62.4% (57.7, 67.1)	53.2% (48.3, 58)	9.2% (6.2, 12.3)	< 0.001*
Four consecutive 24 h recall	59.7% (55, 64.5)	53.2% (48.3, 58)	6.6% (3.9, 9.2)	< 0.001*
Five consecutive 24 h recall	57.5% (52.7, 62.3)	53.2% (48.3, 58)	4.4% (2.2, 6.6)	< 0.001*
Six consecutive 24 h recall	55.6% (50.8, 60.4)	53.2% (48.3, 58)	2.4% (0.7, 4.2)	0.0016*
Recall since birth	50.2% (45.4, 55.1)	53.2% (48.3, 58)	−2.9% (−5.9, 0.1)	0.0455*

*McNemar’s test *p* - value <0.05

The result indicates that compared to the EBF estimates obtained from seven repeated 24 h recall, the EBF estimates from a single 24 h recall overestimated EBF magnitude by 23 percentage points (95% CI 19.2, 27.8). Compared to seven repeated 24 h recall, estimates of EBF prevalence obtained by two repeated 24 h recall overestimated the magnitude of EBF by 13 percentage points (95% CI 9.8, 16.9). Recall since birth resulted in an estimate of EBF that is lower by 2.9 percentage point (95% CI 5.9, 0.1) as compared to seven repeated 24 h recall (Table 1).

When examining difference in EBF prevalence between different age groups, it tends to persistently decrease as the age of the infant’s increases regardless of the methods used (Table 2). The degree of overestimation by single 24 h recall varies across different age groups. Compared to seven repeated 24 h recalls, a single 24 h recall overestimated the magnitude of EBF by 14.4 percentage points (95% CI 6.1, 22.8) among 0–1 month old infants, by 25.6 percentage point (95% CI 17.9, 33.5) among 2–3 month old infants and by 26.4 percentage point (95% CI 19.4, 33.4) among 4–5 month old infants. This overestimation was statistically significant with *p* - value of <0.001. The degree of overestimation in all age groups tends to decrease as the number of observation days increases.

**Table 2:** Patterns of changes in estimate of EBF prevalence among infants aged 0 - < 6 months using single24 h recall, multiple 24 hour recall and recall since birth among different age groups, Butajira, Ethiopia (*n* = 412)

Age group (0–1 months (*n* = 90))				
	EBF Percent (95% CI)	7 days recall	% overestimation (95% CI)	McNemar’s *p -* value
Single 24 h recall	88.9% (82.3, 95.5)	74.4% (65.3, 83.6)	14.4% (6.1, 22.8)	0.0003*
2 days recall	84.4% (76.8, 92.1)	74.4% (65.3, 83.6)	10% (2.7, 17.3)	0.0039*
3 days recall	81.1% (72.9, 89.4)	74.4% (65.3, 83.6)	6.7% (0.5, 12.9)	0.0313*
4 days recall	81.1% (72.9, 89.4)	74.4% (65.3, 83.6)	6.7% (0.5, 12.9)	0.0313*
5 days recall	78.9% (70.3, 87.5)	74.4% (65.3, 83.6)	4.4% (−0.9, 9.8)	0.1250
6 days recall	76.7% (67.8, 85.6)	74.4% (65.3, 83.6)	2.2% (−1.9, 6.3)	0.5
Recall since birth	70% (60.3, 79.7)	74.4% (65.3, 83.6)	−4.4% (−11.6, 2.8)	0.2891
Age group (2–3 months (*n* = 144))				
	EBF Percent (95% CI)	7 days recall	% overestimation (95% CI)	McNemar’s *p -* value
Single 24 h recall	84.0% (78, 90.1)	58.3% (50.2, 66.5)	25.6% (17.9, 33.5)	< 0.001*
2 days recall	73.6% (66.3, 80.9)	58.3% (50.2, 66.5)	15.3% (8.7, 21.8)	< 0.001*
3 days recall	68.1% (60.3, 75.8)	58.3% (50.2, 66.5)	9.7% (4.2, 15.3)	0.0002*
4 days recall	63.9% (55.9, 71.3)	58.3% (50.2, 66.5)	5.6% (1.1, 10)	0.0078*
5 days recall	61.1% (53.1, 69.2)	58.3% (50.2, 66.5)	2.8% (−0.6, 6.2)	0.1250
6 days recall	59.7% (51.2, 67.3)	58.3% (50.2, 66.5)	1.2% (−1.2, 3.9)	0.5
Recall since birth	56.3% (48, 64.5)	58.3% (50.2, 66.5)	−2.1% (−7.3, 3.1)	0.3657
Age group (4–5 months (*n* = 178))				
	EBF Prevalence (95% CI)	7 days recall	% overestimation (95% CI)	McNemar’s *p -* value
Single 24 h recall	64.6% (57.5, 71.7)	38.2% (31, 45.4)	26.4% (19.4, 33.4)	< 0.001*
2 days recall	51.7% (44.3, 59.1)	38.2% (31, 45.4)	13.5% (7.9, 19.1)	< 0.001*
3 days recall	48.3% (40.9, 55.7)	38.2% (31, 45.4)	10.1% (5.1, 15.1)	< 0.001*
4 days recall	45.5% (38.1, 52.9)	38.2% (31, 45.4)	7.3% (2.9, 11.7)	0.0003*
5 days recall	43.8% (36.5, 51.2)	38.2% (31, 45.4)	5.6% (1.7, 9.6)	0.0016*
6 days recall	41.6% (34.3, 48.9)	38.2% (31, 45.4)	3.4% (0.2, 6.6)	0.0313*
RSB	35.4% (28.3, 42.5)	38.2% (31, 45.4)	−2.8% (−7.8, 2.3)	0.2253

*McNemar’s test *p* - value <0.05

## Discussion

This study was conducted in order to measure the degree of overestimation of the prevalence of the EBF rate derived from a single 24 h recall method compared to seven days of 24 h recall data. The highest prevalence of EBF was found (76.7%) when a single 24 h recall was used. The prevalence of EBF decreases as the number of days of recall increased. The prevalence of EBF also tends to persistently decrease as the age of the infant increases.

Our result showed a disagreement in exclusive breastfeeding rate when different methods of recall were used. The highest difference in EBF prevalence was between a single 24 h recall and seven repeated 24 h recall. By taking seven repeated 24 h recall as a reference, a significant overestimation as high as 23.5% in EBF rate was observed when a single 24 h recall was used. Several previous studies also showed disagreements between different methods of recall. By comparing a single 24 h recall with data collected by monthly recall, one study found a 25% overestimation in EBF rate among 1–4 month old infants [19].

According to our result overestimation in EBF rate varied across different age groups of infant. Comparing data obtained from a single 24 h recall with seven repeated 24 h recall the largest over estimation was observed among infants aged 4–5 months (26.4%) followed by 25.6% among 2–3 months old infants and 14.4% among 0–1 month old infants. The disaggregated data show us that the overestimation among infants aged 0–1 month is much lower compared to other age groups but it is still high. This shows that mothers are feeding their infants exclusively only on some days of the week even at young age. According to our data the overestimation among infants aged 2–3 months and 4–5 months differs only slightly (0.8%). This suggest that most mothers who practice giving something other than breast milk early in the infants life tend to carry on with this practice throughout the six month. Similarly a study comparing 24 h recall with data obtained by daily record showed an overestimation of 41% at two months of age, 43% at four months of age and 9.2% at six months of age when a single 24 h recall was used [16]. The overestimation is attributed to the fact that infants who are given food or fluid other than breast milk on irregular basis would be categorized as exclusively breastfed unless they had not received food or fluid in the past 24 h. These irregular feeding practices also tend to be more frequent as the infant gets older. This might be attributed to the fact that traditionally mothers would stay at home for the first few months, therefore they have more time to breastfeed exclusively and to provide the care needed for their child.

Irregular changes in infant feeding are difficult to capture unless there is a continuous assessment of the infant feeding practice. As indicated in our result, by increasing the number of observation days we can increase the possibility of the indicator capturing the day to day variation in infant feeding. By taking seven repeated 24 h recall as a reference and by increasing the number of observation to just two days we can decrease the degree of overestimation to 13.3%. If the observation day goes as high as six days the overestimation will only be 2.4%.

In this study we took the 7th day data as a single 24 h recall estimate and a retrospective consecutive measurement to obtain repeated 24 h recall estimate. The assumption behind this is to simulate whether a single 24 h recall could represent the infants entire feeding practice by showing if the last day of recall was representative of the week’s feeding practice of the infant. If we took the first day as a single 24 h recall estimate the overestimation compared to seven repeated 24 h recall would be by 18.4%. If we took two prospective consecutive measure of 24 h recall as two day estimate the overestimation compared to seven repeated 24 h recall would by 10.1%.

A study done in Boston has recommended for data to be collected longitudinally, when possible, in order to improve accuracy [23]. Even though longitudinal data improves accuracy, the process is costly and time consuming.

An infant can be exclusively breastfed for a period and receive other food due to a change in circumstance and then return to exclusive breastfeeding [24]. This variability can only be detected if the indicator covers a longer duration of the infant’s life rather than a single 24 h. In this study an indicator with this capability was recall since birth. Since it covers a longer duration than the reference method it has led to estimates of EBF that were lower than those obtained from seven repeated 24 h recall (2.9% difference). This difference is therefore due to mothers who have exclusively breastfed their infant in the previous seven days but have given their infant something other than breast milk such as water, juice or semi-solid food at some point in the infant’s life.

Though recall since birth provides a more valid estimate of exclusivity of breastfeeding from birth to the measurement day, the major problem with the use of recall since birth is the potential recall bias. This indicator relies on a mother’s ability to accurately recall whether she has introduced food or fluid other than breast milk. In our study, recall since birth provided a data that was very close with the data obtained from seven repeated 24 h recall (only 2.9% difference). This might be suggestive that recall bias was not a problem in our study even though we cannot rule out the bias for sure.

There were several studies that have reported good agreement between maternal recall and prospective data. One of these studies found out that information obtained retrospectively based on maternal recall can provide accurate estimation of initiation and duration of breastfeeding even 20 years after birth [25]. A study validating maternal recall after six years, revealed that there was no difference between prior and current recall about breastfeeding duration [26]. A systematic review also suggested that maternal recall can provide an accurate estimation of initiation and duration of breastfeeding especially when the recall duration is over a short period (≤ 3 years) [27].

A study comparing seven days recall with trice weekly 48 h recall, showed that recall of infant feeding practices over the previous seven days had high sensitivity and specificity for EBF [21]. In this study recall of feeding practices over the previous seven days reflected the feeding pattern of infants accurately. The authors have suggested that seven days recall method could be used for prospective studies [21]. Similarly in our study we have seen that the prevalence obtained from recall since birth (up to current age) approximated the result obtained from seven repeated 24 h recall. Therefore recall of EBF over the previous week could be an alternative method to assess EBF practice since birth.

From our analysis we have seen that infant feeding practice varies on a daily basis and that using a single 24 h recall to categorize type of infant feeding is misleading. This misclassification is especially dangerous because the potential to further improve EBF rate will not be addressed, thereby overlooking opportunities to advance child health. This indicates the need for an indicator with greater accuracy to estimate EBF than the currently recommended 24-h recall. We suggest the use of single 24 h recall in assessing EBF prevalence should be reconsidered and recommend the use of one week recall (i.e. recall of practice over the previous seven day) as an alternative to single 24 h recall.

We believe that the current methodology has its limitation and that needs to be acknowledged. Since we conducted repeated visits (seven days) with the mothers, it is very difficult to rule out the possibility of social desirability bias in the response to the child feeding questions. Mothers could respond favorably to the breastfeeding practices questions in the subsequent interviews. The strength of this study is the use of itemized check list in order to minimize recall bias during the assessment of recall since birth, the use of randomly selected infants and the use of standardized questionnaire.

## Conclusion

A single 24 h recall method substantially overestimates the prevalence of exclusive breastfeeding among infants aged 0 - < 6 months compared to repeated 24 h recalls over a seven day period. Recall since birth and seven repeated 24 h recall methods gave a comparable EBF prevalence. Therefore, our findings encourage using repeated 24 h recall over a one week period to have a plausible estimate of EBF, in settings where conducting repeated 24 h recall measurements is viable. A recall of infant feeding practices over the previous seven day period might be a valid alternative, although this method was not examined in this study. Based on our findings we recommend policy makers reconsider the use of single 24 h recall in assessing EBF prevalence. We recommend further validation work of the seven days recall method.

## Abbreviations

DSS: Demographic Surveillance System
EBF: Exclusive Breastfeeding
PCA: Principal Component Analysis
WHO: World Health Organization
